# Relevance of Biofilm Models in Periodontal Research: From Static to Dynamic Systems

**DOI:** 10.3390/microorganisms9020428

**Published:** 2021-02-19

**Authors:** María Carmen Sánchez, Andrea Alonso-Español, Honorato Ribeiro-Vidal, Bettina Alonso, David Herrera, Mariano Sanz

**Affiliations:** 1 ETEP (Etiology and Therapy of Periodontal and Peri-Implant Diseases) Research Group, University Complutense of Madrid, 28040 Madrid, Spain; mariasan@ucm.es (M.C.S.); andalons@ucm.es (A.A.-E.); honoribe@ucm.es (H.R.-V.); bettimal@ucm.es (B.A.); davidher@ucm.es (D.H.); 2 Medicine Department, Faculty of Medicine, University Complutense of Madrid, 28040 Madrid, Spain

**Keywords:** oral biofilms, confocal laser microscopy, scanning electron microscopy, biofilm models, dynamic systems, Robbins device, periodontal diseases, peri-implant diseases, dental implants

## Abstract

Microbial biofilm modeling has improved in sophistication and scope, although only a limited number of standardized protocols are available. This review presents an example of a biofilm model, along with its evolution and application in studying periodontal and peri-implant diseases. In 2011, the ETEP (Etiology and Therapy of Periodontal and Peri-Implant Diseases) research group at the University Complutense of Madrid developed an in vitro biofilm static model using representative bacteria from the subgingival microbiota, demonstrating a pattern of bacterial colonization and maturation similar to in vivo subgingival biofilms. When the model and its methodology were standardized, the ETEP research group employed the validated in vitro biofilm model for testing in different applications. The evolution of this model is described in this manuscript, from the mere observation of biofilm growth and maturation on static models on hydroxyapatite or titanium discs, to the evaluation of the impact of dental implant surface composition and micro-structure using the dynamic biofilm model. This evolution was based on reproducing the ideal microenvironmental conditions for bacterial growth within a bioreactor and reaching the target surfaces using the fluid dynamics mimicking the salivary flow. The development of this relevant biofilm model has become a powerful tool to study the essential processes that regulate the formation and maturation of these important microbial communities, as well as their behavior when exposed to different antimicrobial compounds.

## 1. Introduction

Bacterial biofilms can be defined as complex, functionally and structurally organized sessile microbial communities, characterized by a multi-species diversity that synthesize an extracellular, biologically active polymer matrix (exopolysaccharides, EPS), which anchors cells to each other as well as to surfaces [1,2,3]. The term biofilm was originally used in technical and environmental microbiology and it emerged in 1982 after assessing that *Staphylococcus aureus* had formed a biofilm on a cardiac pacemaker lead [4]. These microbial communities have been proven to be ubiquitous in almost every environment [5], being found in a wide variety of both biotic and abiotic surfaces, including living tissues, such as teeth, and medical devices, such as dental implants [6,7]. Furthermore, many infectious diseases affecting humans are related to the formation of biofilms and/or the development of dysbiotic biofilms, which not only can serve as a reservoir and source of pathobionts, but also prevent effective antimicrobial therapeutic strategies [8]. Their importance is highlighted by the increasing number of patients treated with the implantation of medical devices, since once the device has been implanted, there will be a competition between host tissue cells and bacteria to adhere to its surface and, if biofilms are formed, they can be a source of pathogens, or they can be modified to dysbiotic biofilms, and a limitation to antimicrobial therapies [9]. Among these device-related infections, catheter-associated urinary tract infections are among the most common healthcare-associated infections [10].

This ability of bacteria to form biofilms on biotic and abiotic surfaces, as on implanted and indwelling devices, has been studied in clinical and pre-clinical studies, although the intrinsic mechanisms of biofilm formation and maturation remain unclear, and it is still a challenge to solve. For this reason, several in vitro biofilm models have been developed [11,12,13,14,15,16], with the first biofilm models described in the 1970s [17,18]. These single-dimension, steady-state models were important for understanding the mechanisms behind biofilm formation and differentiation and, specifically, studied mechanisms of bacterial attachment, surface-associated spreading and dispersion, bacterial cell-to-cell communication molecules and environmental cues or underlying genetic elements involved in biofilm homeostasis. Nowadays, biofilm modeling has improved in sophistication and scope towards multi-dimensional dynamic models, although, there are no well-established multi-species biofilm models to expand our knowledge on the complex biofilm’s structure and its mechanisms of pathogenicity, as well as for testing antimicrobial and anti-biofilm strategies. Only a limited number of standardized protocols are available [16,19,20,21,22,23] and, among them, some have been widely cited: biofilms formed by opportunistic human pathogens *Pseudomonas putida* and *Pseudomonas aeruginosa* [12,13]; the chronic wound biofilm model with *P. aeruginosa*, *S. aureus* and *Enterococcus faecalis* [14]; and biofilms involving fungi, mainly *Candida* species [15].

This manuscript reports the application of an in vitro biofilm model used in periodontal research and the evolution and improvement of this model to develop representative models of periodontal and peri-implant biofilms.

## 2. Oral Biofilms and In Vitro Biofilm Models

Among oral diseases in which biofilms are their main etiological factor, caries, periodontal and peri-implant diseases are of great importance, since these chronic inflammatory diseases are among the most prevalent human infections worldwide [24,25,26,27,28]. These diseases are associated with dysbiotic complex biofilms formed over the surface of natural and artificial structures within the oral cavity [1,29,30,31]. Due to this complexity, the development and validation of representative and reproducible oral biofilm models have been a challenge; hence, only few in vitro models are available in the scientific literature for studying the structure, formation and maturation of oral biofilms.

In caries research, there is a large variety of in vitro biofilm models, using different bacterial growth technologies, such as cell/tissue culture plates [32,33,34,35,36,37], multi-plaque artificial mouth (MAM) [38], constant depth film fermenter (CDFF) [39], chemostats [40], center for disease control (CDC) biofilm reactor [41], flow chambers and flow cell [42,43,44] (e.g., ten Cate´s Model [44]), artificial mouth computer controlled (AMCC) [45,46,47] or the multi-station continuous-culture biofilm model (MSCBM) [48].

In periodontal research, the study of subgingival biofilms was first attempted by placing inserts of different materials into the periodontal pockets of periodontitis patients [49,50], or by using dispersed subgingival plaque/biofilm, a selection of specific subgingival bacteria in microtiter plate assays or in constant-depth film fermenter systems [51,52,53,54,55]. Among these methodologies, using a defined consortium of bacteria is a more reproducible approach and we can assess the behavior and response of one or more pathogens within an environment similar to what occurs in the oral cavity. Conversely, the biofilm models based on studying a microcosm from the patient’s subgingival plaque, although less reproducible, are more similar to biofilms in vivo and the overall biofilm response to antimicrobial agents can be tested. Depending on the aims of the investigation, different biofilm models should be chosen [56]. Similarly, biofilm models can be grown on a static system, being more reproducible and economical, although they hardly reproduce the physico-chemical conditions of the oral environment. Conversely, the dynamic systems enable a continuous nutrient supply, control of the flow and shear conditions or continuous monitoring of the microenvironment, including temperature and pH, among other factors [57].

All these models were mainly used to evaluate different antimicrobial approaches, but very few provided accurate information on how subgingival biofilm forms and which is the sequence of events that occurs in the maturation of a steady-state biofilm. Among the most cited biofilm model studies on subgingival biofilms, the following should be highlighted: the “Calgary biofilm device” proposed by Ceri et al. (1999), with *Escherichia coli*, *P. aeruginosa* and *S. aureus* [58]; the Wimpenny et al. model (1999) [59]; and the Guggenheim et al. model, with *Actinomyces naeslundii, Veillonella dispar, Fusobacterium nucleatum, Streptococcus sobrinus* and *Streptococcus oralis* growing on sintered circular hydroxyapatite (HA) discs [60]. The study carried out by Zijnge et al., to in vivo describe the architecture of supra- and sub-gingival plaque/biofilm, using fluorescent in situ hybridization (FISH), should also be highlighted, describing the location in vivo of the most abundant species from different phyla and species associated with periodontitis on teeth obtained from periodontal patients. The dominance of *Actinomyces* spp., *Tannerella forsythia, Fusobacterium nucleatum*, Spirochaetes and Synergistetes was demonstrated [61].

### Development of a Static/Dynamic Multi-Species Subgingival In Vitro Biofilm Model

**Figure 1:**
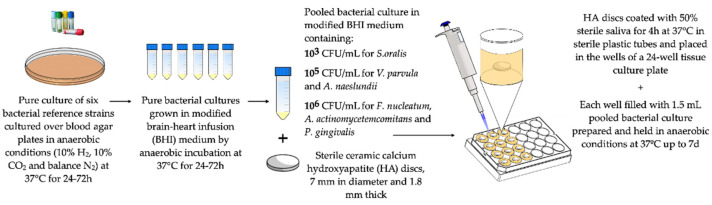
Schematic representation of the Etiology and Therapy of Periodontal and Peri-Implant Diseases (ETEP) research group’s static, in vitro multi-species biofilm model [62]. The selected species represent initial (*Streptococcus oralis* and *Actinomyces naeslundii*), early (*Veillonella parvula*), secondary (*Fusobacterium nucleatum*) and late colonizers (*Porphyromonas gingivalis* and *Aggregatibacter actinomycetemcomitans*). Bacterial concentration is expressed as colony-forming units per milliliter (CFU/mL).

In 2011, the ETEP (Etiology and Therapy of Periodontal and Peri-Implant Diseases) research group at the University Complutense of Madrid, Spain, and the research team of DENTAID S.L. laboratories (Sant Cugat del Vallès, Barcelona, Spain), developed an in vitro biofilm static model using representative bacteria from the subgingival microbiota [62]. The proposed model included six reference strains, representing the initial (*S. oralis* and *A. naeslundii*), early (*Veillonella parvula*), secondary (*F. nucleatum*) and late colonizers (*P. gingivalis* and *Aggregatibacter actinomycetemcomitans*), which were incubated over sterile ceramic calcium hydroxyapatite (HA) discs coated with sterile saliva within the wells of presterilized polystyrene tissue culture plates [62] (Figure 1).

The formed biofilms were collected at different times to study their structure and spatial distribution with confocal laser scanning microscopy (CLSM), demonstrating higher percentages of vital microorganisms in the z-axis towards the central and upper layers of the biofilm, when compared with the deeper layers and areas adjacent to the HA disc surface in steady-state biofilms (72-h biofilms). This differential vitality distribution had been previously described using histochemical and electron microscopy studies [63]. Furthermore, the biofilm bacterial kinetics were studied using the Terminal Restriction Fragment Length Polymorphism (T-RFLP) analysis [62], demonstrating a pattern of bacterial colonization and maturation similar to what has been described in the development of the subgingival biofilms in vivo, with a sequential colonization from the initial and early to secondary and late colonizers, as the structure of the biofilm became well-defined and mature [62].

This model has also been replicated by other research groups with different applications, such as the study of the effect of shock waves on multi-species oral biofilms [64], or the in vitro effect of replacing amoxicillin with penicillin V, when used in combination with metronidazole, in a biofilm model [65].

The research team of DENTAID S.L. laboratories, together with the ETEP research group, evolved the static model to a dynamic one [66], growing bacteria under flow and shear conditions with higher similarity to the actual oral cavity conditions. This dynamic model used a bioreactor, a Robbins device (where the HA discs bathed in saliva were placed) and a flow system, which allowed the growth of up to 7-day-old biofilms. This model resulted in the formation of highly reproducible multi-species oral biofilms, reproducing the biofilms’ physiological, structural, biochemical and molecular characteristics. In addition, this system was proven appropriate for assessing antimicrobial activity [66].

## 3. Antimicrobial Strategies Evaluated in In Vitro Biofilm Models

The emerging advent of bacterial resistance to commonly used antimicrobial agents, and the inherent capacity of mature biofilms to resist antimicrobial agent penetration and activity, have prompted clinicians and researchers to seek for innovative antimicrobial compounds [67,68]. Furthermore, it is well established that that antimicrobial testing in planktonic bacterial growth provides significantly different results than when the same antimicrobial agents are tested in biofilm models [69,70,71]. There is, therefore, a priority to develop well-validated, multi-species in vitro biofilm models to test these emerging molecules, as a prior step to clinical studies, to demonstrate their potential enhanced capabilities to penetrate biofilms, without eliciting development of bacterial resistance.

Although conventional culture techniques have been the laboratory reference method for evaluating the antimicrobial effect of antiseptics or antibiotics, the use of molecular techniques has become more and more relevant [72]. However, one disadvantage with the use of molecular methods to identify and quantify bacteria is their inability to distinguish between viable and dead microorganisms [72,73]. To overcome this problem, our research group set up a testing method using quantitative polymerase chain reaction (qPCR) combined with the use of a discriminating dye, propidium monoazide (PMA), for the selective quantification of viable periodontal pathogens after antimicrobial treatments, both in the planktonic and under biofilm phenotypes [74,75]. The efficacy of PMA for differentiating viable and dead *P. gingivalis*, *A. actinomycetemcomitans* and *F. nucleatum* cells was demonstrated; indeed, this PMA-qPCR method has been frequently used for testing the effect of antimicrobial agents in in vitro oral biofilm models [74,75].

Combining both qPCR and PMA treatment, and applying the static multi-species in vitro biofilm model described above, the antimicrobial activity of three commonly used antiseptics in mouth rinse formulations were tested: 0.12% chlorhexidine (CHX) and 0.05% cetylpyridinium chloride (CPC) without alcohol, a combination of four essential oils (EOs; thymol 0.06%, eucalyptol 0.09%, methyl salicylate 0.06% and menthol 0.01%) without alcohol and 0.05% CPC without alcohol [76]. The results demonstrated the ability of the tested compounds in controlling biofilm growth and maturation, corroborating the antimicrobial efficacy that the listed agents have shown in clinical studies (randomized clinical trials) [77]. However, in spite of this positive effect, their long-term use has been associated with secondary effects, such as irritation of the mucous membranes, tooth staining or increases in calculus formation, which has prompted clinicians and researchers to seek for new antimicrobial compounds, such as natural products capable of inhibiting the proliferation or adhesion of bacterial pathogens in the mouth without eliciting any secondary effects [77,78,79,80].

Within this research line, our research group has focused on evaluating polyphenols, since their application had previously shown inhibitory activity on the growth of different *Streptococcus* spp. and other bacteria [81,82], and there was scarce information about its effect in multi-species biofilm models and, specifically, with bacteria relevant to oral conditions [82,83]. When tested with the described static biofilm model, red wine and oenological extracts showed a moderate antimicrobial activity, with statistically significant reductions in the total bacterial counts and in counts of *A. actinomycetemcomitans, P. gingivalis* and *F. nucleatum*, although modest when compared with regularly used antiseptics, which demonstrated reductions of, at least, 2–3 log in the bacterial counts [84]. Similarly, cranberry extracts, rich in polyphenols, which have also shown antimicrobial effects, besides their proven anti-oxidative and anti-inflammatory activity [85,86,87], had not often been studied in multi-species, in vitro biofilm models. Our research group studied the antibacterial effect of cranberry compounds using the in vitro, multi-species, static biofilm model, concluding that cranberry extracts demonstrated a relevant anti-biofilm effect, by affecting bacterial adhesion during the first 6 h of biofilm development [88].

Using the same model, we also investigated the antimicrobial activity of two omega-3 fatty acids (PUFAs), docosahexaenoic acid (DHA) and eicosapentaenoic acid (EPA). Previous studies have already shown the double potential activity of PUFAs, due to antimicrobial activity against Gram-positive and Gram-negative bacteria [89,90,91], and due to their proven anti-oxidative and anti-inflammatory effects [92]. With the use of qPCR, CLSM and Scanning Electron Microscopy (SEM), both DHA and EPA demonstrated statistically significant reductions in all the tested bacterial strains included in the model. Besides, structural damage was evidenced by SEM in some of the observed bacteria. Hence, it was concluded that both DHA and EPA (at a dosage of 100 μM) have significant antimicrobial activity against the six bacterial species included in the validated biofilm model [93]. 

There are other examples of the application of in vitro oral biofilms models to test antimicrobial compounds or new antimicrobial strategies, such as the use of the Guggenheim´s biofilm model to evaluate the anti-biofilm activity of photodynamic therapy [94], the combination of photodynamic therapy with nanoparticles [95], the combination of natural antimicrobial compounds with oral probiotics [96], or the applications of cationic antimicrobial peptides (CAMPs), in search of anti-biofilm agents as alternatives to conventional antibiotics and antiseptics [97]. These CAMPs are isolated from plants, animals, bacteria and fungi, and are important elements of host innate immunity [98,99]. The mechanism of action is based on disrupting the bacterial plasma membrane, leading to cell lysis [100]. With regards to periodontal pathogens, CAMPs have been reported to reduce the growth of *P. gingivalis*, in vitro [101].

## 4. Evaluation of New Delivery Formats of Oral Antiseptics Using In Vitro Biofilm Models

Another innovative application of the oral biofilm models has been the search for new vehicles/delivery formats to improve the efficacy of antimicrobial compounds. One of these strategies has been the use of biocompatible non-resorbable nanoparticles, with high local bioactivity, capable of the slow release of well-known antibacterial compounds [102,103]. PolymP-n Active nanoparticles (NPs) have recently shown potential as an adjunct to regenerative therapies [104,105], and due to their surface characteristics, they can be doped with metal cations and antibiotics, capable of eliciting antimicrobial activity and long-term inhibition of bacterial growth [106,107,108,109].

Using the in vitro, multi-species static biofilm model described earlier, we have studied the antibacterial effect of polymeric NPs, doped with different substances: zinc, calcium, silver and doxycycline [110]. It was concluded that coating surfaces with nanoparticles and metallic ions significantly reduced the viability of the mature biofilm and weakened the attachment between the matrix and the early colonizers [110].

A similar strategy has been the use of resorbable, bioactive polymeric nanostructured membranes (NMs), which, if doped with zinc, calcium and doxycycline, could exert antimicrobial activity. Our research group evaluated this effect comparing the experimental NMs, with and without being doped with doxycycline, calcium and zinc. As the positive control, commercially available dense polytetrafluoroethylene (d-PTFE) membranes were used, and as negative controls, we used the HA discs without any membrane. Doxycycline-doped NMs resulted in statistically significant reductions in bacterial load, as evaluated by qPCR and SEM, using the ETEP in vitro, static oral biofilm model [111].

In addition, using the same in vitro biofilm model, our research group studied the antibacterial activity of a new ceramic nanocomposite biomaterial (A2 O3/Ce-TZP) sandblasted with white corundum, coated with two types of antimicrobial glasses, 35ZnO-G and G3-GC glass biocides. Biofilms were grown on the different surfaces and evaluated after 12, 24, 48 and 72 h of incubation by means of CLSM and qPCR combined with PMA. Differences in biofilm formation were detected among the different tested biomaterials: the ceramic material with ZnO-enriched glass biocide demonstrated a clear antibacterial effect at different times of incubation, when compared with the control surface and the other tested biomaterial surfaces, which may represent a good candidate for further testing in dental implant applications [112].

## 5. Transcriptomic Analysis and Protein Differential Expression in Biofilm Growth Using In Vitro Biofilm Models

It is well established that the pathogenicity of oral pathogens may vary depending on their phenotype, being enhanced when these bacteria grow in biofilms, when compared with their growth in planktonic state. Several transcriptomic studies have studied the gene expression profiles of pathogenic bacteria growing in biofilms [113,114,115,116]. Whiteley et al. [115] reported that about 1% of the genes from *P. aeruginosa* demonstrated a differential expression when growing in biofilms, as compared with the planktonic state. Liu et al. [114] studied the phenotype of *Clostridium acetobutylicum* when growing in a biofilm and reported that 16.2% of their genes were differentially expressed in biofilm growth, mainly upregulation of genes involved in amino acid biosynthesis, sporulation, extracellular polymer degradation and other various metabolic processes. Similarly, transcriptomic studies have reported that approximately 18.0% of the W50 genome of *P. gingivalis* was differentially expressed when growing in biofilms [113].

**Figure 2:**
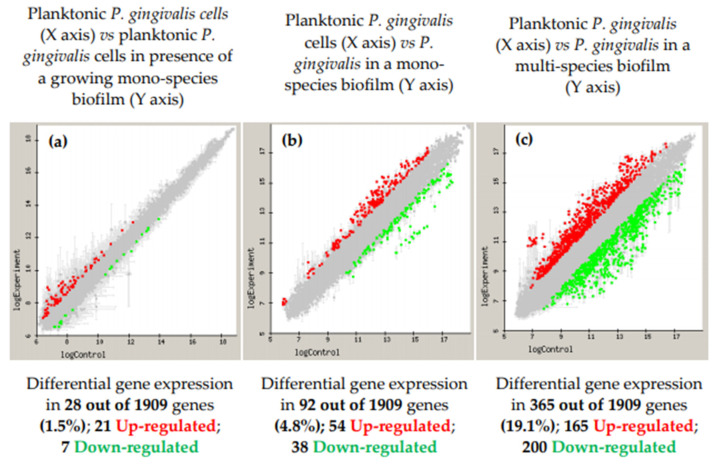
Microarray-based comparative transcriptome (represented in log10) of *Porphyromonas gingivalis* ATCC 33277 in three different conditions. (**a**) Planktonic cells either in the presence of a growing biofilm (test) or in the absence of a biofilm (control); (**b**) mono-species biofilm (test) as opposed to planktonic cells (control); and (**c**) *P. gingivalis* ATCC 33277 expression when growing in a multi-species biofilm (test) compared to its planktonic growth (control). Control planktonic cell gene expression (X-axis) is plotted against the test cells (Y-axis), with a 1.5-fold change (up or down) (**a**) and 2.0-fold change (up or down) (**b**,**c**), with a *p*-value < 0.05. Upregulated genes in the test condition were represented in red and the downregulated genes were colored in green.

However, the knowledge on the gene expression of well-known periodontal pathogens growing in the biofilm state is still very scarce. With the purpose of increasing this knowledge, our research group has carried out a series of transcriptomic studies to evaluate the differential gene expression of periodontal pathogens growing in the in vitro biofilm model with some modifications. The first study evaluated the periodontal pathogen *P. gingivalis* under a planktonic phenotype in the presence or not of a growing mono-species biofilm and showed that 28 of their genes (1.5%) were differentially expressed (upregulated or downregulated) (Figure 2) [117]. Subsequently, the differential gene expression was tested, comparing planktonic versus biofilm growth [118]. A total of 92 out of 1909 genes (4.8%) were differentially expressed by *P. gingivalis* growing in biofilms. Out of those 92 genes, 54 were upregulated, mainly related to cell envelope, transport and binding, or outer membrane proteins, which demonstrated that *P. gingivalis*, when growing in biofilms, changes its virulence profile [118]. Finally, the differential expression of *P. gingivalis* when growing in an in vitro, multi-species biofilm, compared to growing in a planktonic state, was studied [119]. When growing within the multi-species biofilm, 19.1% of the *P. gingivalis* genes were significantly and differentially expressed (165 genes were upregulated and 200 downregulated), exhibiting an increased expression of virulence factors and antioxidant enzymes, especially Hsp proteins and several proteases [119] (Figure 2).

With regard to differential protein expression, studies were carried out with *A. actinomycetemcomitans* comparing its protein expression when growing on biofilms versus the planktonic state [120]. Eighty-seven proteins were differentially expressed during biofilm growth, with 13 over-expressed and 37 down-expressed. The over-expressed proteins were outer membrane proteins (OMPs) and highly immunogenic proteins, which may present candidate virulence factors [120]. In the same line, but for *F. nucleatum* [121], a total of 68 proteins were differentially expressed during biofilm growth, 20 being down-expressed, belonging to metabolism and biosynthesis, and 31 were over-expressed, involved in transcription, metabolism and translation. In addition, in biofilm growth, six out of the seven enzymes that take part in the synthesis of butyrate were differentially expressed, confirming that this metabolic pathway is important in the formation of biofilms of *F. nucleatum* and in its pathogenicity, either in the oral cavity or in other locations of the body [121].

## 6. Study of the Impact of the Material Surface and Topography of Dental Implants on Peri-Implant Biofilm Development

Among implanted medical devices, dental implants to replace missing teeth are extensively used worldwide, and with long-term survival rates between 82% and 99%, as reported in many clinical studies [122,123]. However, successfully osseointegrated implants may frequently suffer complications, mainly the advent of peri-implant inflammatory diseases, a consequence of bacterial contamination and formation of biofilms adhering to the implant surfaces [31]. Peri-implant diseases have been recently defined and classified as peri-implant mucositis and peri-implantitis [30].

Dental implants and their restorative components are sterile when placed in the oral cavity, but once exposed to the oral environment they can become colonized by bacteria. These exposed surfaces bathed in saliva can therefore form biofilms in a similar manner than dental surfaces [124]. The establishment of a stable mucosal seal around the implant necks will serve as a barrier, similar to the gingival tissues, for the ingrowth of bacteria or their products. However, the presence of mature complex biofilms, resulting from defective oral hygiene practices, may lead to chronic inflammation of the peri-implant tissues (peri-implant mucositis). If this condition is left untreated, susceptible individuals may develop bone destruction around the affected implants (peri-implantitis), eventually causing implant loss due to the progression of the bone destruction around the implant [125]. It has been reported that the microbiota associated with peri-implantitis lesions is similar to that observed in periodontitis [126]. However, the micro-surface topography of dental implants is more conducive for biofilm formation than that of tooth surfaces; thus, scientific attention has been placed on studying the role of implant surface micro-roughness in enhancing bacterial biofilm formation around dental implants [124].

This line of research started looking at bacterial adhesion and growth of oral bacteria on titanium (and other materials) discs with different surface characteristics [127,128,129,130,131,132,133], demonstrating a direct correlation between rugosity and bacterial adherence. However, most of the published studies have used simple, in vitro biofilm models, using one—or two—bacterial species and relatively short growth periods (maximum 24 h). These studies, therefore, lack the possibility of studying the biofilm dynamics during maturation and hence their pathogenic potential [134,135]. These limitations can be overcome with the use of mature multi-species biofilms. In some studies, saliva collected from volunteers has been used as substrate to develop biofilms; however, the resulting biofilms lack reproducibility [135] and result in high variability in the microbial patterns, probably reflecting the microbial heterogeneity among individuals in saliva [136].

### 6.1. Static Multi-Species In Vitro Biofilm Model Over Implant-Material Discs

The first approach from our group used the in vitro static biofilm model, to compare biofilm formation on discs of different implant materials and with different micro-rugosity. Results showed statistically significant differences in biofilm thickness and three-dimensional structure of the biofilms, depending on the studied surface (HA, titanium or zirconium) [137]. SEM observations revealed that surface microtopography conditioned biofilm structure after 72 h of incubation. On titanium discs, bacterial cells formed compact bacterial communities, similar to honeycombs in their tridimensional architecture, while on zirconium discs, biofilms were thinner and bacteria formed networks, in which *F. nucleatum* cells formed the core, while the other micro-colonies adhered to them [137]. Differences in biofilm formation may be justified by the increased relative roughness and hydrophobicity of the tested titanium surface, which is intended to improve implant osseointegration, but may also ease the development of complex biofilms [127,138]. A hypothesis derived from the previous observations (differences in the biofilm structure formed over surfaces with different roughness or materials) was also tested, namely, if those differences in the biofilms were associated with differences in their response to commonly used antiseptics; no statistically significant differences were observed [76].

### 6.2. A Static, Multi-Species, In Vitro Biofilm Model Over Whole Dental Implants

The in vitro biofilm model using implant material discs, although evaluating the impact of the micro-surface topography on the biofilm formation, could not assess the impact of the complete implant macro-structure, usually made of threads, combining peaks, valleys and ramps, where biofilms could have differential growth dynamics. To evaluate this, a static, multi-species biofilm model on whole implants was set-up [139,140]. The use of this model revealed the existence of different patterns of bacterial colonization and biofilm formation also dependent on the implant macro-surface topography. Furthermore, the observed cell viability was different depending whether the formed biofilm was located at the peak of the threads, harboring more live bacteria, or at the bottom of the valleys, with a significant increase in dead cells, which indicated that the biofilm mass was affected by the different microenvironments dependent of the implant macro-structure [139]. Using this model, we also showed that implants with a moderately rough surface accumulated significantly greater biomass and a higher number of pathogenic species when compared to implants with minimally rough surfaces [140].

### 6.3. Development of an In Vitro Multi-Species Dynamic Model to Study the Impact of Dental Implant Surface and Topography on Bacterial Biofilms

**Figure 3:**
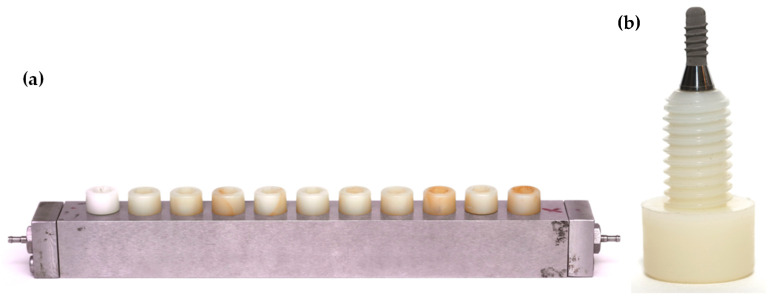
(**a**) Robbins device hosting the (**b**) nylon anchoring screws that carry the implants.

Static oral biofilm models may be of great relevance in the study of different aspects related to oral health, as we have previously described, but they are lacking something important, since they do not resemble the changes in the environmental conditions that occur within the oral cavity, where the forming biofilms are subject to constant physio-chemical changes, dependent on the salivary and crevicular fluid flow. Besides, other authors have highlighted the differences between biofilms growing in static versus dynamic models based on control of the flow rate, shear forces, pH or temperature [141]. For these reasons, an evolution of the static model was developed aiming to overcome these limitations with regard to peri-implant diseases studies. The proposed in vitro dynamic oral biofilm model was based on a modification of the dynamic model described by Blanc et al. [66]: the Robbins device (Figure 3) was de novo designed to host the implants in order to study bacterial deposition, considering both the macro- and micro-structure characteristics of the implants.

**Figure 4:**
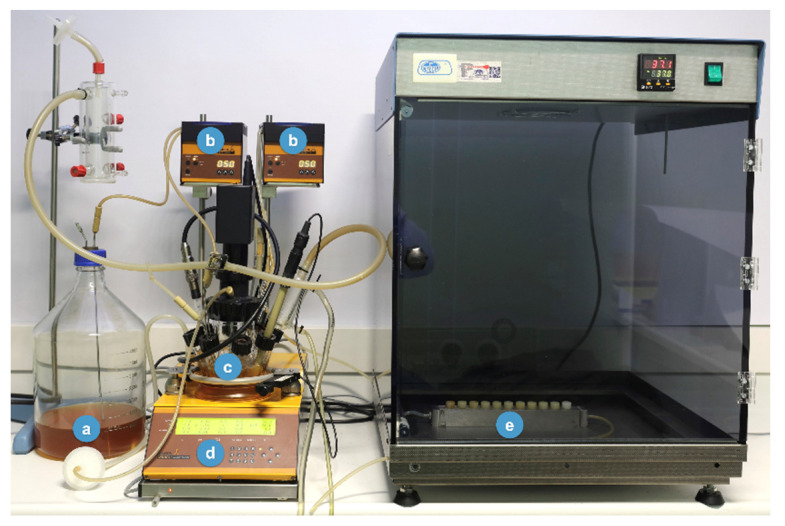
New developed model to generate biofilms over implants. (**a**) Culture medium—brain heart infusion (BHI); (**b**) peristaltic pumps; (**c**) incubation recipient; (**d**) bioreactor (temperature control, pH, pO_2_, agitation and weight); (**e**) Robbins device hosting the implants.

The dynamic open system consists of a series of different components, including, firstly, a sterile recipient (Figure 4a), where the liquid culture medium, specifically modified brain heart (BHI) medium, also used in the previously described static model [62], is pumped to the bioreactor by a peristaltic pump at constant pressure (Figure 4b). The bioreactor (Lambda Minifor^©^ bioreactor, LAMBDA Laboratory Instruments, Sihlbruggstrasse, Switzerland) (Figure 4) will keep the culture medium under controlled conditions, i.e., at 37 °C, pH 7.2 and an anaerobic atmosphere (Figure 4c,d), and will allow for the inoculation of the bacterial mixture. The bacterial mixture of six bacterial strains and the growing conditions used were already described for the static biofilm model [62]. Once inoculated in the bioreactor (Figure 4c), the bacterial mixture is incubated for 12 h, to allow acclimatization of bacteria and then, through a peristaltic pump and under continuous flow (30 mL/h) (Figure 4b), transferred to the Robbins device (Figure 4e). The Robbins device contains the sterile implants under anaerobic conditions (10% H_2_, 10% CO_2_ and the balance N_2_), at 37 °C, placed in a laboratory stove to keep the temperature of the system controlled. Figure 3 shows the implants mounted on the Robbins device with their implant surfaces located within the channel where the bacterial mixture flows during the experimental period, thus allowing the biofilm formation over the whole surface of the implants.

**Figure 5:**
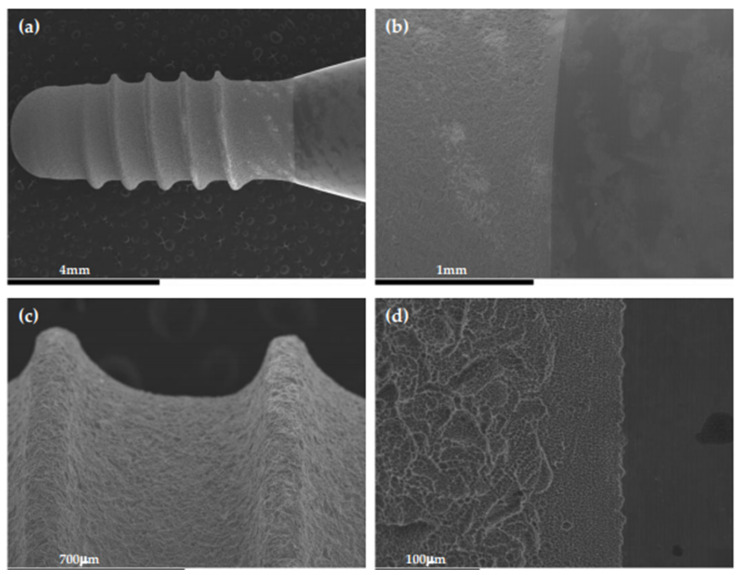
Scanning electron microscopy (SEM) of a Straumann^®^ Tissue Level Standard implant before biofilm formation. Magnification: (**a**) 12×; (**b**) 50×; (**c**) 80×; (**d**) 330×.

This model allows the simultaneous study of different dental implants, with different designs and surfaces. In the present publication, preliminary results of a study carried out with the described open dynamic model are presented [142]. Figure 5 depicts a commercially available dental implant (Institute Straumann AG, Basel, Switzerland) of 8 mm in length and 3.3 mm in diameter, containing the patented sand-blasted and acid-etched moderately rough surface (SLA), used to set-up this model. The macro- and micro-surface topography of this dental implant, when sterile, is depicted in Figure 5.

**Figure 6:**
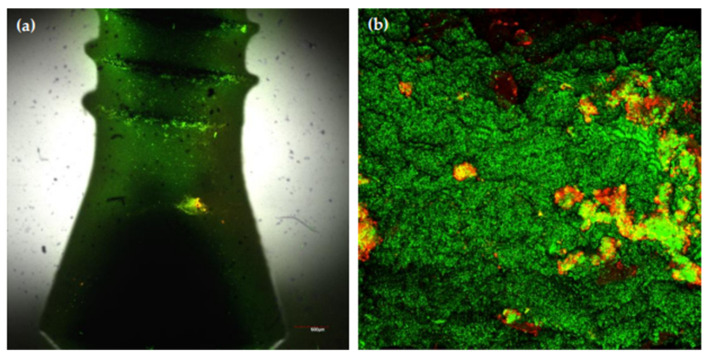
Closer view of (**a**) the coronal area of the Straumann^®^ Tissue Level Standard implant; (**b**) confocal laser scanning microscopy (CLSM) of the 96 h biofilms on the implant surfaces. Specimens were stained with the LIVE/DEAD^®^ BacLight^TM^ Bacterial Viability Kit solution (Molecular Probes B. V., Leiden, The Netherlands) containing SYTO9 and propidium iodine nucleic acid stains. Cells that were dead or dying bacteria were stained in red (PI) whereas cells with an intact membrane were stained in green (SYTO9).

Once bacterial cells were allowed to colonize these implants, confocal laser scanning microscopy at 96 h of incubation demonstrated the surface of the implants covered by live bacteria (in green), forming a discontinuous layer of cells where cells were grouped in “towers” and stacks containing these multi-species, following the characteristic biofilm morphology (Figure 6b) where vital bacteria predominated over dead ones (in red) (Figure 6b). Furthermore, microcolonies were mostly concentrated over the lateral surfaces and the pitch of the threads (Figure 6a).

**Figure 7:**
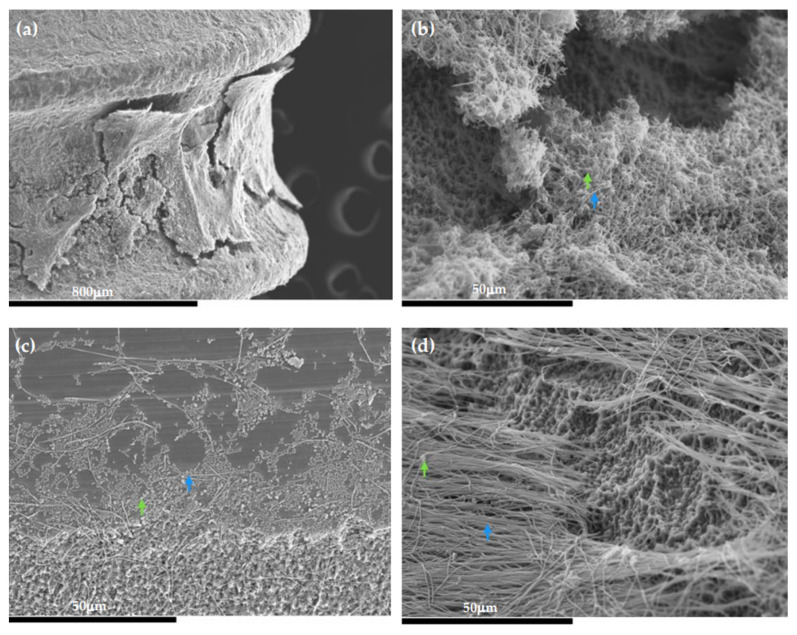
Scanning electron microscopy (SEM) images of the 96-h biofilms over the Straumann^®^ Tissue Level Standard implant. (**a**) View of the biofilm formation between the implant threads; (**b**–**d**) presence of spindle-shaped rods forming three-dimensional structures, which was recognized as *F. nucleatum* (blue arrows), with short streptococcal chains adhering, which was identified as *S. oralis* (green arrows), surrounded by a dense extracellular matrix covering the entire surface.

Figure 7 depicts the morphology of the 96-h biofilms covering the implant surface (Figure 7a) obtained by SEM. These bacterial communities formed stacks (growing masses of bacterial cells) with broad channels among them (Figure 7b). It was noteworthy the presence of spindle-shaped rods forming three-dimensional structures, which was recognized as *F. nucleatum* (Figure 7b,d, blue arrows), with short streptococcal chains adhering, which was identified as *S. oralis* (Figure 7b,d, green arrows), surrounded by a dense extracellular matrix covering the entire surface.

**Figure 8:**
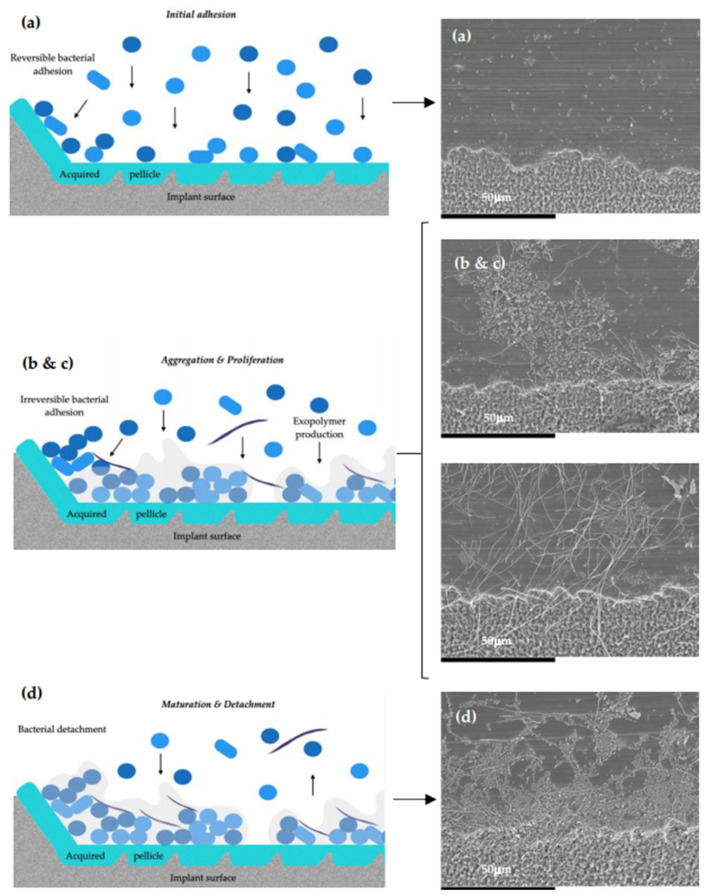
Diagram and scanning electron microscopy (SEM) images after (**a**) 24 h; (**b**) 48 h; (**c**) 72 h; (**d**) 96 h. (**a**) The primary colonization of the implant surface, covered with a pellicle composed mainly by host-derived salivary glycoproteins, occurred, where the bacteria, mainly streptococcal forms, were identified in aggregates over the surface. (**b**,**c**) Depicts cell proliferation and aggregation of new bacteria that occurred, leading to the development of microcolonies, where spindle-shaped rods are identified, suggestive of *F. nucleatum*, which forms networks with the adhered microcolonies of bacteria. (**d**) The formation of heterogenous and three-dimensional bacterial structures, characterized by larger masses of sessile microorganisms, separated from each other by channels and interstitial spaces. Magnification: (**a**–**d**) 1000×.

When studying biofilm formation in the dynamic open system by SEM, the development process of the multi-species biofilm showed, firstly, how the primary colonization of the implant surface, covered with a pellicle composed mainly by host-derived salivary glycoproteins, occurred, where the bacteria, mainly streptococcal forms, were identified in aggregates over the surface (Figure 8a). Next, the cell proliferation and aggregation of new bacteria occurred, leading to the development of microcolonies, where spindle-shaped rods were identified, suggestive of *F. nucleatum*, which formed networks with the adhered microcolonies of bacteria (Figure 8b,c). Thereafter, heterogenous and three-dimensional bacterial structures were formed, characterized by larger masses of sessile microorganisms, separated from each other by channels and interstitial spaces (Figure 8d).

The described biofilms, formed on implant surfaces in the dynamic biofilm model, corroborate previous descriptions using the static biofilm model [137,139,140] and confirm earlier characterizations of the biofilm dynamics made on HA discs using the static biofilm model [66]. This dynamic biofilm model is based on two key components: the bioreactor, where the six species can grow planktonically, and the Robbins device, where multi-species biofilms are formed over the implant surfaces when the bacterial mixture is pumped through a continuous culture with a flow of 30 mL/h, which may resemble the unstimulated salivary flow in the mouth [143]. Nonetheless, the described in vitro dynamic multi-species biofilm model has clear advantages, since the physico-chemical conditions are closer to those found in the oral cavity. Furthermore, this biofilm model growing on the whole implant allows not only the study of the biofilm growth depending on the micro surface (mainly different roughness), but also the macro-structure of the implant, studying the biofilms differentially growing at the peaks of the threads versus the slopes or the valleys between threads. Furthermore, biofilms formed over whole implants enable the study of different antimicrobial and disinfection strategies when applied to the surface of the implant. These studies cannot be done in biofilm static models growing on disks. In addition, the possibility of using custom-made Robbins devices, adapted for different specimens (dental implants or other devices or materials), allows the development of complex biofilms in closer conditions to the clinical scenarios.

## 7. Conclusions

Biofilm modeling has improved in sophistication and scope, although only a limited number of standardized and reproducible protocols are available. This publication presents an example of a biofilm model, with its evolutions and applications in studying periodontal and peri-implant diseases. The evolution of in vitro multi-species biofilm models started from the mere observation of biofilm growth and maturation on static models over hydroxyapatite or titanium discs, to the development of complex dynamic systems, aiming to reproduce the ideal microenvironmental conditions for bacterial growth within a bioreactor, and reaching the target surfaces using fluid dynamics mimicking the salivary flow. The development of effective and reproducible biofilm models provides powerful tools to study the essential processes that regulate the formation and maturation of these important microbial communities, as well as their behavior when exposed to different antimicrobial compounds.

## Data Availability

Not applicable.
